# Validation of multiparametric MRI by histopathology after nephrectomy: a case study

**DOI:** 10.1007/s10334-020-00887-9

**Published:** 2020-09-20

**Authors:** Anneloes de Boer, Tobias T. Pieters, Anita A. Harteveld, Peter J. Blankestijn, Clemens Bos, Martijn Froeling, Roel Goldschmeding, Hans J. M. Hoogduin, Jaap A. Joles, Bart-Jeroen Petri, Marianne C. Verhaar, Tim Leiner, Tri Q. Nguyen, Arjan D. van Zuilen

**Affiliations:** 1 Department of Radiology, University Medical Center Utrecht, Utrecht University, Utrecht, The Netherlands; 2 Department of Nephrology and Hypertension, University Medical Center Utrecht, Utrecht University, Utrecht, The Netherlands; 3 Department of Pathology, University Medical Center Utrecht, Utrecht University, Utrecht, The Netherlands; 4 Department of Vascular Surgery, University Medical Center Utrecht, Utrecht University, Utrecht, The Netherlands

**Keywords:** Multiparametric magnetic resonance imaging, Histology, Kidney, Kidney transplantation

## Abstract

**Objectives:**

Renal multiparametric MRI (mpMRI) is a promising tool to monitor renal allograft health to enable timely treatment of chronic allograft nephropathy. This study aims to validate mpMRI by whole-kidney histology following transplantectomy.

**Materials and methods:**

A patient with kidney transplant failure underwent mpMRI prior to transplantectomy. The mpMRI included blood oxygenation level-dependent (BOLD) MRI, *T*_1_ and *T*_2_ mapping, diffusion-weighted imaging (DWI), 2D phase contrast (2DPC) and arterial spin labeling (ASL). Parenchymal mpMRI measures were compared to normative values obtained in 19 healthy controls. Differences were expressed in standard deviations (SD) of normative values. The mpMRI measures were compared qualitatively to histology.

**Results:**

The mpMRI showed a heterogeneous parenchyma consistent with extensive interstitial hemorrhage on histology. A global increase in *T*_1_ (+ 3.0 SD) and restricted diffusivity (− 3.6 SD) were consistent with inflammation and fibrosis. Decreased T_2_ (− 1.8 SD) indicated fibrosis or hemorrhage. ASL showed diminished cortical perfusion (− 2.9 SD) with patent proximal arteries. 2DPC revealed a 69% decrease in renal perfusion. Histological evaluation showed a dense inflammatory infiltrate and fibrotic changes, consistent with mpMRI results. Most interlobular arteries were obliterated while proximal arteries were patent, consistent with ASL findings.

**Discussion:**

mpMRI findings correlated well with histology both globally as well as locally.

**Electronic supplementary material:**

The online version of this article (10.1007/s10334-020-00887-9) contains supplementary material, which is available to authorized users.

## Introduction

Kidney transplantation is the treatment of choice for patients with end-stage kidney disease [1]. Close monitoring of the graft is crucial for early detection of treatable conditions [2]. Traditional methods of graft monitoring like the measurement of plasma creatinine and proteinuria only deviate when the majority of nephrons are irreversibly lost. Some centers use protocol biopsies to detect subclinical disease. However, transplant biopsies are invasive, carry the risk of sampling errors and are associated with a small risk of graft loss [3]. Therefore, protocol biopsies are not standard of care in all centers and are not suitable for monitoring disease progression. Multiparametric kidney MRI may help overcome these problems. MRI is sensitive to various relevant functional and structural markers like perfusion, oxygenation and fibrosis [4]. It can map the kidneys as a whole and is non-invasive, making it a suitable candidate for monitoring of local and global disease progression. For example, *T*_1_ and the apparent diffusion coefficient (ADC) correlate with fibrosis on histology and could be used to predict current graft dysfunction and future eGFR decline [5–7]. Perfusion measurements can be performed without contrast agent using arterial spin labeling (ASL) and have been shown to correlate with reduced capillary density [7–9].

Several histological findings in protocol biopsies, such as interstitial fibrosis and tubular atrophy (IF/TA) and capillary rarefaction, strongly correlate to graft survival [10, 11]. Both capillary rarefaction and IF/TA may be caused by a treatable condition, such as subclinical rejection or return of the original disease.

In our center, a multiparametric renal MRI protocol has recently been developed and evaluated [9]. The protocol consists of relaxometry (*T*_1_ and *T*_2_ mapping), BOLD MRI, DWI, ASL and 2D phase contrast (2DPC) to measure renal blood flow (RBF). Normative values are available in 19 healthy volunteers [9].

Previous studies on correlation of MRI measures with histology used kidney biopsies for histological validation, but given the often heterogeneous distribution of damage, biopsies might not be representative for the entire kidney. We recently had the unique opportunity to perform multiparametric MRI in a patient scheduled for allograft explantation, which allowed for a comparison of MRI findings to whole-kidney histology and to evaluate MRI performance in identifying intra-organ heterogeneity. The objective of this study was to validate the comprehensive MRI protocol to whole-kidney histology using (1) a comparison of MRI findings to normative values in healthy controls and (2) a comparison between whole-kidney histology and local as well as global changes in MRI measures.

## Case description

The patient was a 46-year-old female with an extensive history of liver fibrosis and end-stage kidney disease of unknown origin. Eleven years ago, she underwent a kidney transplantation with a kidney donated after circulatory death. Several creatinine rises in the following years were attributed to tacrolimus toxicity and acute rejection. Seven years post-transplantation, the patient started hemodialysis because of chronic rejection, likely caused by non-adherence to the immunosuppressive regimen. Eleven years post-transplantation, chronic hematuria and onset of new HLA-antibodies suggested ongoing rejection for which she was treated with prednisolone. A urologic analysis did not suggest other causes for hematuria. Because of persistent pain in the region of the transplant kidney and persistent hematuria, it was decided to explant the allograft. During surgery, the kidney had to be cut from the surrounding capsule which led to minor superficial lesions and hemorrhaging.

Prior to surgery, informed consent for inclusion in an ongoing study on multparametric MRI in transplant kidneys was obtained and the MRI was scheduled 10 days before transplantectomy.

## Materials and methods

### MRI acquisition and processing

The MRI examination was performed on a 3T MR system (Ingenia, Philips Healthcare, Eindhoven, the Netherlands; software release 5.3.1). The patient was asked to avoid salt- and protein-rich meals and to drink 2 L per 24 h of non-alcoholic liquids on the day of the scan to roughly standardize hydration and dietary conditions. The scan protocol, MR system and scan conditions were the same as described earlier [9]; therefore, the baseline results of that study could be used as normative values.

The scan protocol is described in detail elsewhere [9]. In short, it consisted of localizer images followed by an anatomical *T*_1_-weighted Dixon. BOLD/$R_{2}^{*}$ mapping was performed with a 15-echo gradient echo sequence. Diffusion-weighted imaging was performed with a set of *b* values and directions per *b* value that allowed for both a DTI and an IVIM analysis. *T*_1_ mapping was performed with a slice-cycled inversion recovery sequence and *T*_2_ mapping was achieved using *T*_2_ preparations. Single slice 2D phase contrast allowed for quantification of blood velocity in the (transplant) renal artery. ASL was performed using a flow-attenuated inversion recovery scheme with four different inversion times. Total scan time was approximately 1 h. Scan parameters are presented in Table 1.

**Table 1:** Overview of scan parameters

	*T*_1_w Dixon	*T*_1_ map	$T_{2}^{*}$/BOLD	*T*_2_ map	DTI/IVIM	FAIR-ASL	M_0_ images	2D PC
Sequence	3D GE	2D GE	2D GE	2D GE	2D SE	2D GE	2D GE	2D GE
Fast imaging	–	EPI	–	EPI	EPI	EPI	EPI	TFE
Echoes	2	1	15	1	1	1	1	1
TE1;ΔTE (ms)	3.5;1.1	22	4.6;4.6	21	62	22	22	3.3
TR (ms)	7.5	6500	213	5000	3247	6500	6500	5.5
FA (°)	8	90	25	30	90	90	90	10
Half-scan	–	–	–	–	0.8	–	–	–
Orientation	Coronal oblique	Coronal oblique	Coronal oblique	Coronal oblique	Coronal oblique	Coronal oblique	Coronal oblique	Sagittal oblique
Slices	35	11	14	7	20	5	5	1
Voxel size (mm)	1.5 × 1.5 × 3	3 × 3 × 6	3 × 3 × 3	3 × 3 × 6	3 × 3 × 3	3 × 3 × 6	3 × 3 × 6	1.5 × 1.5 × 6
FOV (mm)	320 × 400 × 70	244 × 244 × 76	360 × 360 × 48	244 × 244 × 48	336 × 336 × 60	244 × 244 × 34	244 × 244 × 34	320 × 250
Recon matrix	400	96	240	96	112	96	96	256
Parallel imaging factor	2	1.5	3	1.5	2.1	1.5	1.5	3
Acquisition time (mm:ss)	00:19	01:12	5 × 00:09	03:37	02:42	4 × 02:23	00:33	2 × 00:10
Respiratory compensation	Breath-hold	Synchronized breathing	Breath-hold	Free breathing	Free breathing	Synchronized breathing	Synchronized breathing	Breathhold
Remarks	Dixon recon of water only images	Cycled multi-slice inversion recovery sequence; TIs: 55–2035 with 198 ms steps	–	MLEV preparation with composite 180° block pulses with *T*_2_ weightings 15, 48.75, 82.5, 116.25, 150 ms	*b* values 0, 10, 20, 50, 100, 200, 500	TIs 800, 1400, 2000, 2600 ms; 10 label-control pairs per TI	4 averages	25 phases, venc 150 cm/s, exact acquisition time depends on heart rate

*BOLD* blood oxygenation level dependent, *DTI/IVIM* diffusion tensor imaging/intravoxel incoherent motion, *FAIR-ASL* flow-attenuated inversion recovery arterial spin labeling, *2DPC* 2D phase contrast, *TE* echo time, *GE* spoiled gradient echo, *EPI* echo planar imaging, *TR* repetition time, *FA* flip angle, *FOV* field of view, *TI* inversion time

Processing involved post hoc motion correction, fitting of the appropriate model and delineation of regions of interest (ROIs), avoiding areas affected by artifacts. Quality of all scans was assessed visually by an expert reader (AB, 5 years of experience in renal imaging) and scans of insufficient quality as assessed by visual inspection were excluded from analysis. In the patient, no discrimination between cortex and medulla was possible. Therefore, whole-parenchyma ROIs were used to allow for comparison between the patient and controls. ROI generation was semi-automated, using a combination of thresholding and k-means clustering [9]. For ROI generation in the graft, manual intervention was required since the normal anatomy was virtually lost. The collecting system could be identified on the anatomical scans and was excluded. The remaining parenchyma was entirely included. For detailed information on post-processing, see [9].

After processing, several parameter maps were obtained. For relaxometry, *T*_1_, *T*_2_ and $R_{2}^{*}$ maps were generated. For the DWI data, both diffusion tensor imaging (DTI) and intravoxel incoherent motion (IVIM) analysis were performed. DTI analysis yielded the fractional anisotropy (FA) and the mean diffusivity (MD). IVIM enabled the measurement of the contribution of microvascular perfusion (perfusion fraction, PF) to the diffusion coefficient (*D*). Local perfusion was measured with ASL MRI using four different inversion times, allowing both quantification of perfusion and determination of arterial transit times (ATT). 2D phase contrast (2DPC) MRI was used to measure total blood flow through the renal artery.

From the $R_{2}^{*}$ map (BOLD) and the *T*_2_ map, $R_{2}^{\prime }$ could be calculated:

$${R}_{2}^{^{\prime}}={R}_{2}^{*}-\frac{1}{{T}_{2}},$$

where $R_{2}^{\prime }$, $R_{2}^{*}$ and *T*_2_ are the median values of the parenchymal ROIs. Compared to $R_{2}^{*}$, $R_{2}^{\prime }$ is considered to reflect oxygenation more directly since it is not influenced by changes in *T*_2_.

### Histology

After fixation in formalin, coronal sections of the explanted kidney were obtained from the upper pole (ventral, dorsal and lateral side), lower pole (ventral, dorsal and lateral side) and hilar region (ventral and dorsal side). In addition, we added a nephrectomy sample from a tumor nephrectomy where we show the healthy tissue as comparison to the explanted graft. Sections were paraffin embedded. Next, slides were cut at 3 µm and stained with hematoxylin and eosin (H&E), periodic acid–Schiff (PAS) and methenamine silver (Jones’ stain) using standard protocols. Finally, slides were analyzed by two experienced renal pathologists (RG, TQN).

### Statistical analysis

MRI measures of the entire parenchyma were not normally distributed due to substantial differences between cortex and medulla in healthy kidneys. Therefore, median values and interquartile ranges (IQR) were calculated for the entire parenchyma ROI analysis. To assess changes in texture, local standard deviation (SD) maps were calculated where each voxel contains the SD of the 3 × 3 surrounding voxels. The median values within the ROI on these local SD maps were reported as well. For healthy subjects, the groupwise mean and SD were reported for all measures. Differences between the patient’s values and normative values were expressed as percentage differences with respect to the normative values and in SDs of the normative values. Since only one patient was analyzed, no comparative statistical tests were performed.

To compare the voxel distribution within the parameter maps between healthy and the diseased kidney, histograms were used. For each examination in each subject, a histogram was calculated. In the controls, the median histogram was calculated. Parameter variations within controls were demonstrated by the IQR, the 10–90 and 2.5–97.5 inter-percentile ranges, and are shown as shaded areas on the histograms. Results of histology were described qualitatively. The comparison between histopathology and MRI findings was performed in a qualitative way by an experienced renal pathologist (TQ) and an author with 5 years of experience in renal MRI (AB).

## Results

Data of 19 healthy subjects with a median age of 49 (IQR 45–57) were used as normative values. The patient tolerated the MRI examination well and all data were of sufficient quality to be included in the current analysis.

### MRI—comparison to normative values

**Fig. 1:**
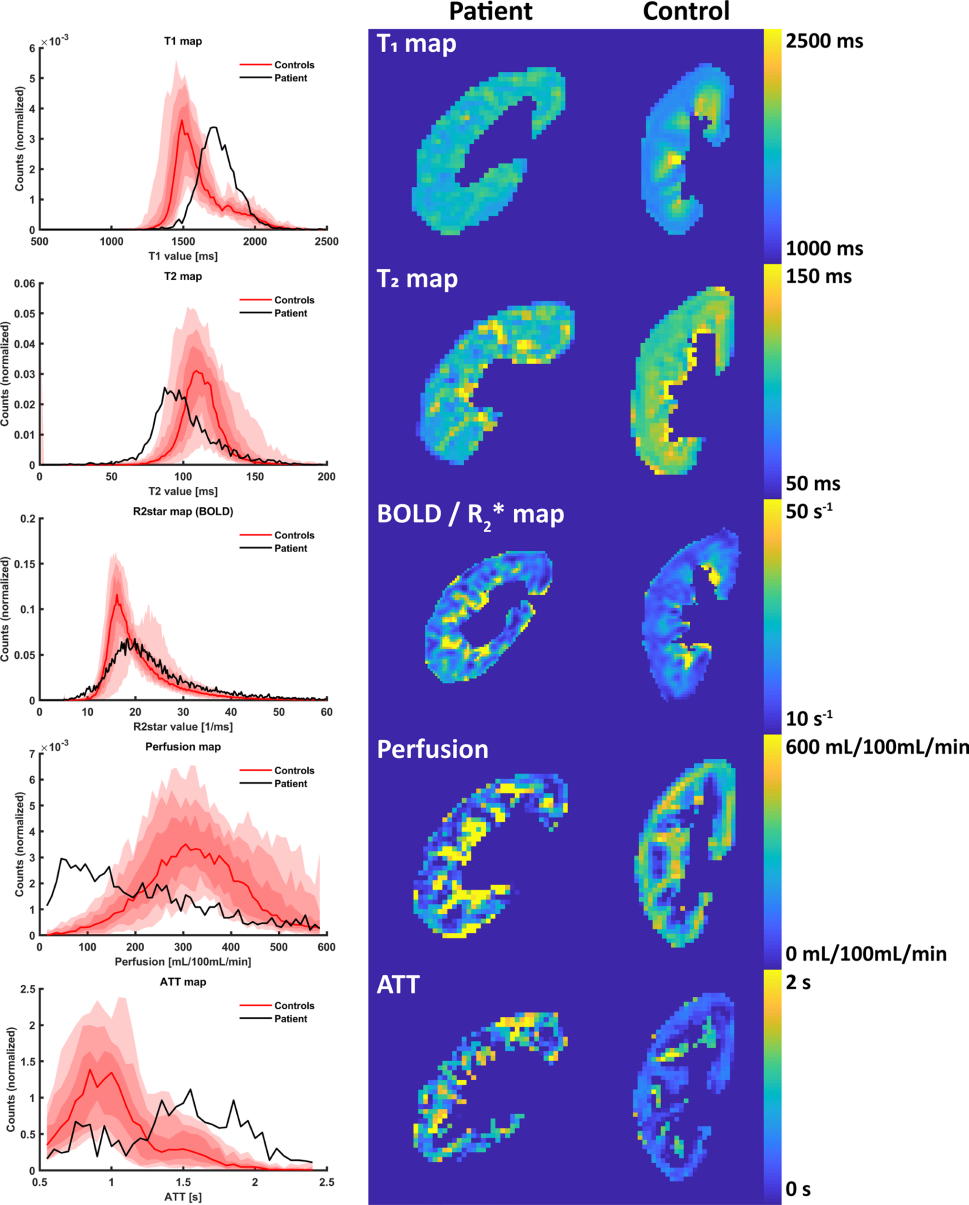
Histograms (left) and parameter maps for the patient (middle column) and a single control (right) for *T*_1_ and *T*_2_ mapping, BOLD and perfusion and ATT as assessed by ASL. The histograms show the voxel distribution in the patient and the median voxel distribution in controls, along with the spread between controls. The shaded areas denote the interquartile range, the 10–90 and the 2.5–97.5 interpercentile range (dark to lightly shaded). The histograms are normalized. *BOLD* blood oxygenation level-dependent MRI, *ATT* arterial transit time, *ASL* arterial spin labeling

**Fig. 2:**
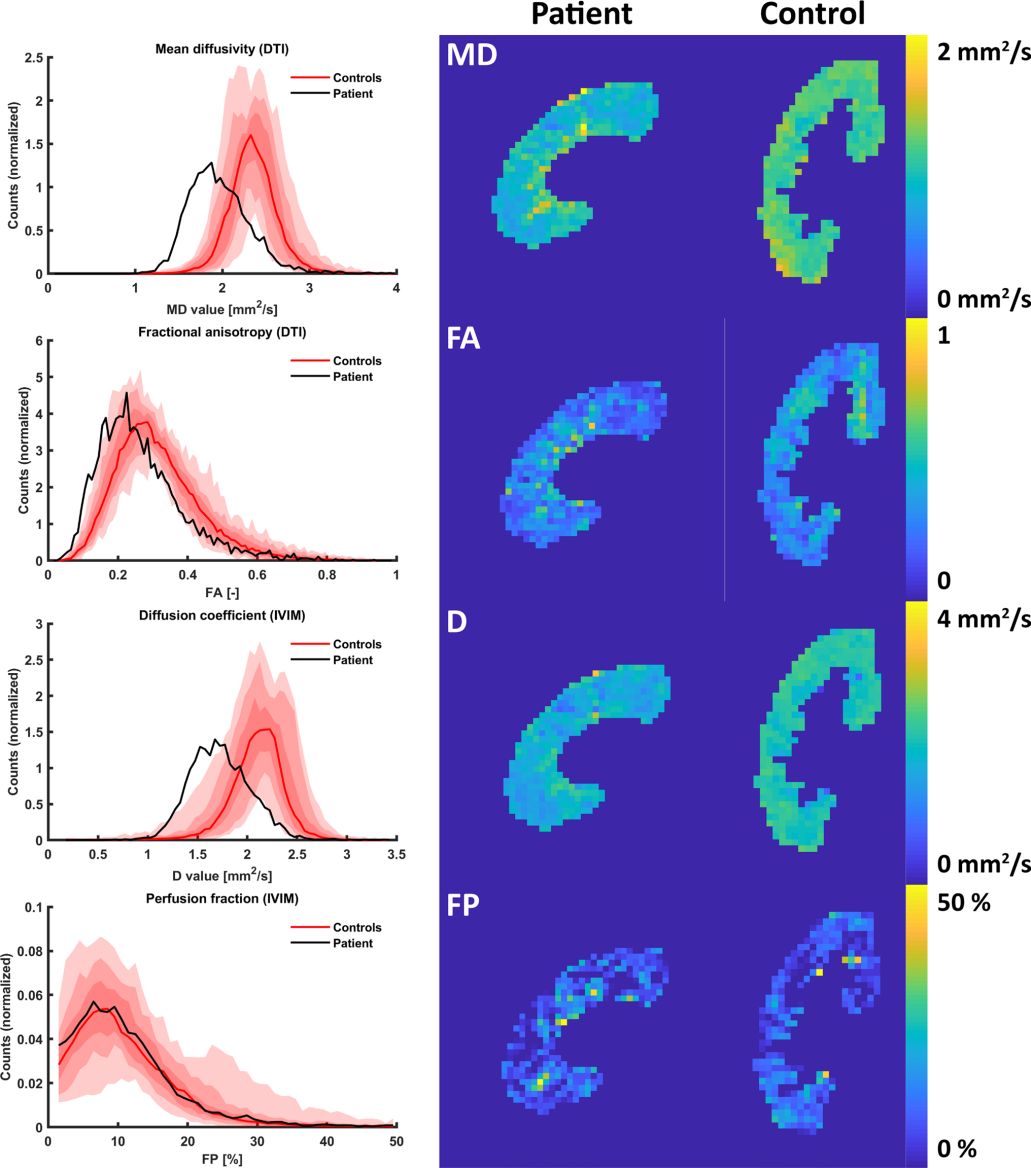
Histograms (left) and parameter maps for the patient (middle column) and a control (right) for all diffusion measures: MD and FA from the DTI analysis and D and PF from the IVIM analysis. The histograms show the voxel distribution in the patient and the median voxel distribution in controls, along with the spread between controls. *MD* mean diffusivity, *FA* fractional anisotropy, *D* diffusion coefficient, *PF* perfusion fraction

In Figs. 1 and 2, the MRI parameter maps belonging to the patient are shown alongside corresponding native kidney maps of a single control. For almost all MR measurements, noticeable differences could be appreciated between the patient and the control.

In healthy subjects, the cortex and medulla could be easily discriminated thanks to the higher *T*_1_ in the medulla. Within the cortex and medulla, the *T*_1_ was rather homogeneous. In the patient, however, there was no difference between cortex and medulla and the *T*_1_ values within the parenchyma were heterogeneous (Fig. 1). However, due to the loss of corticomedullary differentiation, this was not reflected in the IQR (167 ms vs 213 (37) ms, Table 2) nor in the local SD (91 vs 96 (16) ms). Overall, *T*_1_ was increased in the diseased kidney due to microstructural changes in the tissue, most likely fibrosis and influx of inflammatory cells (Table 2).

**Table 2:** MRI measures for the patient compared to controls

	Patient	Controls	Difference (%)	Difference (SD)
*T*_1_ map (ms)				
Median	1732	1556 (59)	+ 11	+ 3.0
IQR	167	213 (37)	− 21	− 1.2
Local SD	91	96 (16)	− 5	− 0.3
*T*_2_ map (ms)				
Median	97	113 (8)	− 14	− 1.8
IQR	25	15 (3)	+ 67	+ 3.9
Local SD	14.5	7.8 (1.3)	+ 87	+ 5.1
$R_{2}^{*}$ map/BOLD (s^−1^)				
Median	21.7	19.0 (2.1)	+ 15	+ 1.3
IQR	10.8	7.1 (1.4)	+ 52	+ 2.5
Local SD	3.9	2.4 (0.4)	+ 61	+ 4.0
$R_{2}^{\prime }$ (BOLD and *T*_2_ map) (s^−1^)				
Median	11.4	10.1 (1.9)	+ 13	+ 0.7
ASL–perfusion (ml/100 mL/min)				
Median	170	339 (59)	− 50	− 2.9
IQR	359	140 (35)	+ 155	+ 6.3
Local SD	172	79 (45)	+ 118	+ 2.1
ASL–ATT (s)				
Median	0.93	0.48 (0.15)	+ 95	+ 3.1
IQR	0.99	0.43 (0.15)	+ 128	+ 3.7
Local SD	0.45	0.20 (0.13)	+ 122	+ 2.0
2DPC–blood flow (ml/min)				
NA	140	422 (159)	− 67	− 1.8
DTI–MD (mm^2^/s)				
Median	1.9	2.4 (0.1)	− 19	− 3.6
IQR	0.46	0.31 (0.06)	+ 48	+ 2.5
Local SD	0.26	0.19 (0.04)	+ 34	+ 1.8
DTI–FA (fraction)				
Median	0.23	0.29 (0.03)	− 20	− 1.9
IQR	0.15	0.15 (0.02)	+ 1	+ 0.1
Local SD	0.10	0.09 (0.01)	+ 1	+ 0.1
IVIM–*D* (mm^2^/s)				
Median	1.7	2.1 (0.1)	− 20	− 2.9
IQR	0.42	0.30 (0.07)	+ 41	+ 1.8
Local SD	0.23	0.17 (0.03)	+ 31	+ 1.5
IVIM–PF (%)				
Median	7.9	8.8 (3.3)	−9	− 0.3
IQR	10.5	10.3 (3.1)	+ 1	0
Local SD	5.6	5.2 (1.2)	+ 8	+ 0.3

Median values, interquartile ranges and the local SDs of whole-parenchyma ROIs are reported. For the controls, the groupwise mean (standard deviation) of the ROI medians, IQRs and local SDs are calculated. Percentage difference between patient and controls with respect to the controls was calculated, as well as the absolute difference in units of the SD of the distribution in healthy volunteers

*IQR* interquartile range, *SD* standard deviation, *BOLD* blood oxygenation level-dependent MRI, *ASL* arterial spin labeling, *AT* arterial transit time, *2DPC* 2D phase contrast, *DTI* diffusion tensor imaging, *MD* mean diffusivity, *FA* fractional anisotropy, *IVIM* intravoxel incoherent motion, *D* diffusion coefficient, *PF* perfusion fraction

The parenchymal *T*_2_ of the diseased kidney was decreased as compared to controls (Table 2). In Fig. 1, marked heterogeneity was seen throughout the parenchyma reflected by large increase in both local SD (14.5 vs 7.8 (1.3) ms) and IQR (25 vs 15 (3) ms) in the diseased kidney. Regionally, *T*_2_ approached 150 ms, which is the upper cut-off value of the fit. *T*_2_ values in this range indicate high water content, for example, edema or fluid collections. Due to fixation, this could not be confirmed histologically.

The BOLD or $R_{2}^{*}$ map confirms the finding of increased heterogeneity, which was also reflected in the broader histogram compared to the controls (Fig. 1), the increased IQR (10.8 vs 7.1 (1.4) s^−1^) and the marked increase in local SD (3.9 vs 2.4 (0.4) s^−1^). No marked difference in global median $R_{2}^{*}$ value between the patient and healthy subjects was seen (Table 2).

**Fig. 3:**
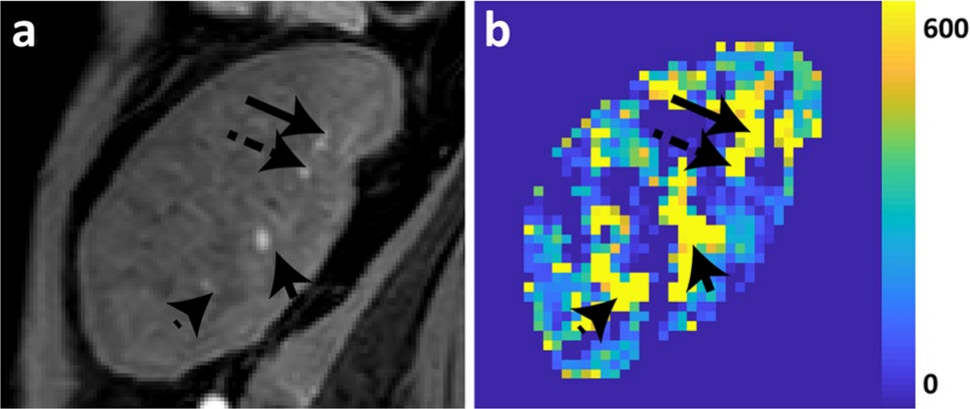
Comparison of anatomical *T*_1_-weighted Dixon (**a**) to perfusion map as obtained by ASL (**b**). In the Dixon, vessels have high signal thanks to inflow of fresh blood. On the perfusion map, vessels can be recognized as areas with very high flow (~ 600 mL/100 mL/min). Because the spatial resolution of the Dixon is much higher compared to the perfusion map (1 × 1 × 2 mm for the Dixon compared to 3 × 3 × 6 mm for the perfusion map), the vessels seem larger and more extensive on the perfusion map. Arrows show the locations of corresponding vessels on the Dixon and on the perfusion map

Regarding perfusion, in healthy subjects, areas with high perfusion were located in the cortex, but in the patient, these areas were located more centrally, corresponding to the locations of large vessels (Fig. 3). Around patent large vessels, perfusion was very high, while other areas seemed devoid of any blood supply. In healthy subjects, the latter was only seen in the inner medulla. The arterial transit times also showed a heterogeneous pattern in the diseased kidney (IQR 0.99 vs 0.43 (0.15)), compared to homogeneous and relatively short arrival times in the healthy cortex. Overall, 2DPC showed a marked decrease in blood flow through the renal artery in the diseased kidney (140 vs 422 (159) mL/min).

Results of the diffusion analysis are shown in Fig. 2. Both MD and D showed higher restriction of diffusion compared to healthy subjects (Table 2), consistent with fibrosis and inflammation. A slight increase in heterogeneity was seen in the patient in all diffusion measures. FA was slightly decreased indicating less anisotropy, while no clear difference was seen between the patient and the controls for the PF. However, it should be noted that the quality of PF map was lower than the other parameter maps.

### Histology—comparison to MRI

**Fig. 4:**
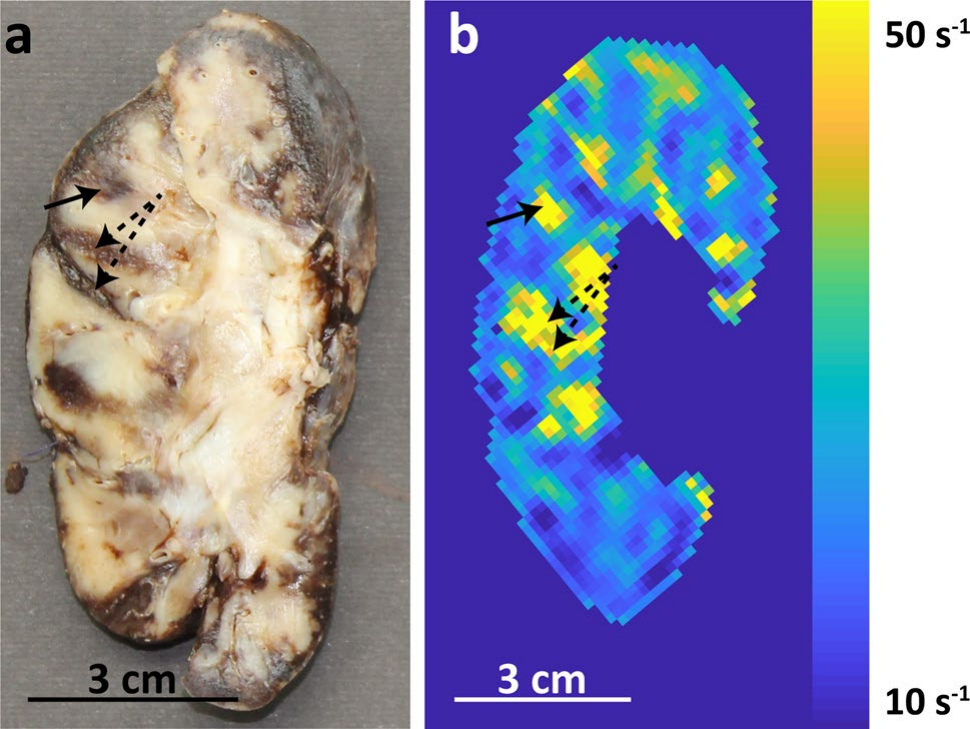
Macroscopic image of the explanted kidney (**a**) shown alongside the corresponding $R_{2}^{*}$ map (**b**). The $R_{2}^{*}$ map clearly shows the hemorrhages which are denoted by the arrows. The characteristic shape of the hemorrhage at the dashed arrows can also be recognized on the $R_{2}^{*}$ map

Macroscopically, the explanted kidney was small (10 × 4.5 × 4.5 cm) and multiple hematomas were seen (Fig. 4a). Hematomas are expected to be visible at $R_{2}^{*}$ (BOLD) maps as areas with high $R_{2}^{*}$. The areas with $R_{2}^{*}$ close to 50 s^−1^ (the cut-off value) seen on the $R_{2}^{*}$ map correlated with the locations of hematomas (Fig. 4a, b).

**Fig. 5:**
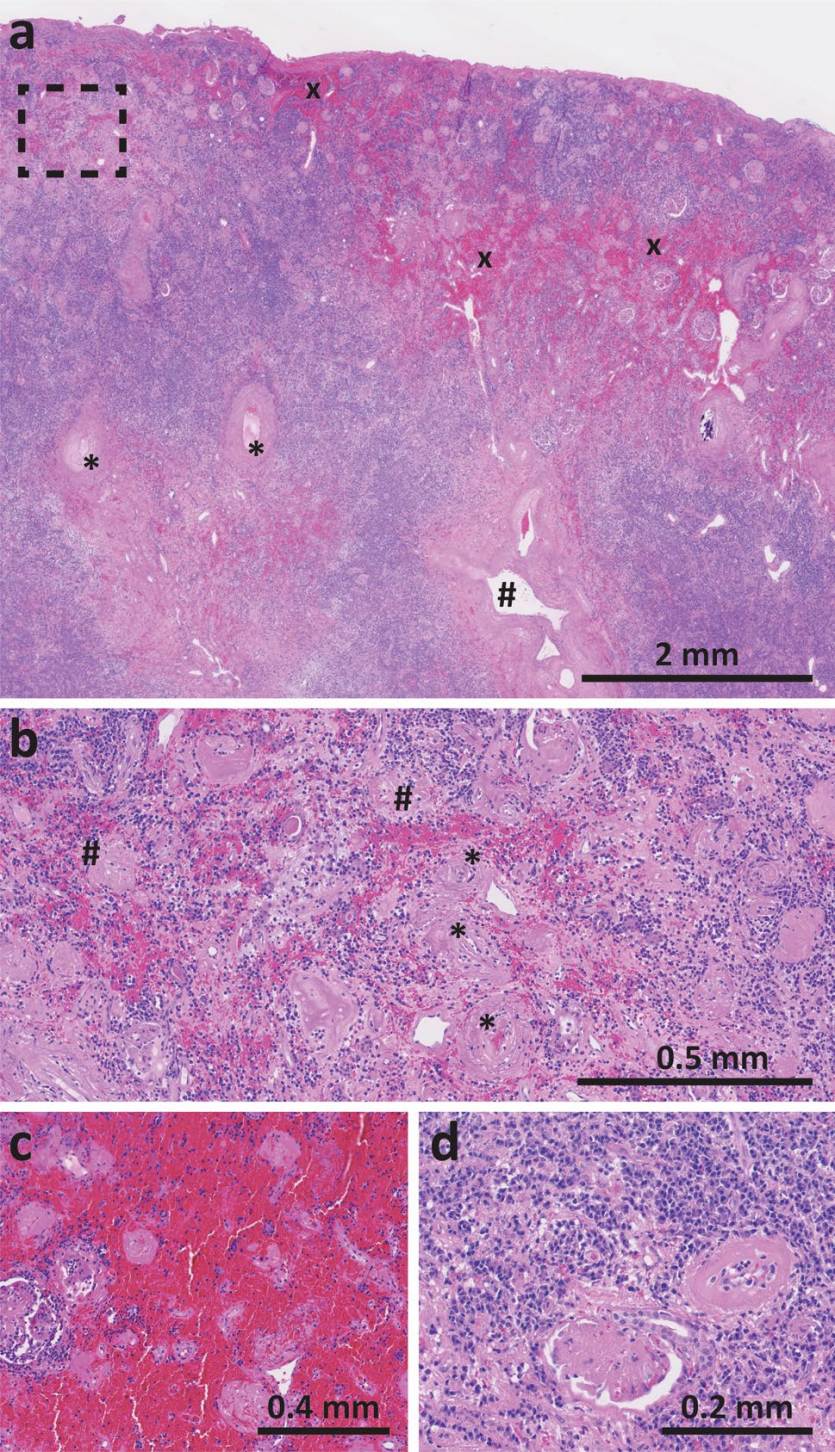
Histology findings of the explanted kidney (all H&E stain); **a** overview of cortex and medulla. The medulla contains patent interlobar arteries (#) and arcuate arteries (*). Regions affected by hemorrhaging are denoted with an ×; **b** higher magnification of cortex annotated by the dashed rectangle in A showing completely obliterated interlobular arteries (*) and globally sclerosed glomeruli (#); **c** cortical area with extensive interstitial hemorrhage; **d** cortical area with dense interstitial inflammatory infiltrate

Microscopic examination revealed extensive microstructural damage as compared to healthy histology (Supplemental Fig. 1). Most proximal vasculature, including the interlobar and arcuate arteries in the medulla, was still patent (Fig. 5a), while the distal vasculature including the interlobular arteries in the cortex were severely obliterated due to concentric intimal fibrosis with infiltration of plasma cells and lymphocytes (Fig. 5b). This was consistent with the perfusion maps obtained from ASL, which showed diminished perfusion of the parenchyma with very high perfusion around large vessels (Fig. 1).

Throughout the kidney but particularly at the dorsal side of the upper pole and lateral side, extensive areas with interstitial hemorrhages were seen (Fig. 5c). Most erythrocytes within these hemorrhages were intact and showed properties consistent with acute hemorrhage, although some scattered areas with hemosiderin-laden macrophages were found, indicative of older hemorrhage. The cortex was affected by severe glomerulosclerosis, tubular atrophy and interstitial fibrosis, and contained a dense inflammatory infiltrate consisting mainly of plasma cells (Fig. 5d). The medulla displayed a similar severity of inflammation. Although some non-sclerosed glomeruli were present focally, atrophy, inflammation and hemorrhage affected almost all proximal tubules. Those global changes in the renal parenchyma were in line with the MRI findings. These showed increased *T*_1_ and restricted diffusion consistent with inflammatory infiltrate and fibrosis and a decreased *T*_2_ which is consistent with fibrosis and hemorrhages.

## Discussion

In this study, we aimed to validate multiparametric MRI of a kidney transplant with end-stage disease to whole-kidney histology obtained after explantation. Multiparametric MRI findings were compared to normative values and to histology. Important clinical predictors of graft survival, IF/TA and capillary rarefaction could be measured with MRI. IF/TA resulted in restriction of diffusion as detected by a decrease in MD and D and in an increase in *T*_1_. Capillary rarefaction could be identified by reduced parenchymal perfusion, which was detected as a decrease in total perfusion by ASL and 2DPC, as well as by a radical shift in perfusion distribution on ASL perfusion maps.

The combination of fibrosis and inflammation in the allograft parenchyma was reflected by increased *T*_1_ and diffusion restriction [12, 13]. On the other hand, *T*_2_ was decreased, probably due to the severe fibrosis [14, 15] and the scattered hemorrhages since dominance of the widespread inflammation would have been expected to result in increase of *T*_2_ [16].

Global changes in median $R_{2}^{*}$ and $R_{2}^{\prime }$ were minimal. However, heterogeneity of $R_{2}^{*}$ as measured with the local SD was markedly increased (+ 4.0 SD compared to controls). This probably was caused by the widespread fresh hemorrhages that caused local increases in $R_{2}^{*}$ values. While the hemorrhages also increased $R_{2}^{*}$ and $R_{2}^{\prime }$ on a global level, this was compensated by a decrease in $R_{2}^{*}$ (and $R_{2}^{\prime }$) due to capillary rarefaction which reduced blood volume and perfusion. Diminished oxygen demand in the virtually nonfunctioning kidney decreased deoxyhemoglobin synthesis as well, decreasing $R_{2}^{*}$ [17]. However, this was compensated by the oxygen demand of active inflammatory cells.

Perfusion of the diseased kidney was less than half of that measured in control subjects as demonstrated by both ASL and 2DPC. The large increase in median ATT indicated that arterial blood supply to the renal parenchyma was heavily delayed. Perfusion maps showed diminished parenchymal perfusion with maintained perfusion in the large vessels. This was consistent with histology showing patent interlobar and arcuate arteries, whereas the most distal interlobular arteries and arterioles were obliterated.

The obliteration of smaller vessels would have been expected to result in a decreased PF as measured with DWI [5]. In repeatability studies in healthy volunteers and patients, large variations in repeat measurements of PF have been observed [5, 9, 18–20]. The lack of difference in PF in might, therefore, be attributable to an unreliable measurement, which makes it currently less suitable for diagnostic purposes.

Changes in most MRI measurements in the diseased transplant kidney correlated well with pathological changes seen at macroscopic as well as microscopic analysis of the explanted allograft. Since the diseased allograft had been nonfunctioning for years prior to MRI and explantation, not all results of this study can be simply extrapolated to predict and interpret MRI findings in less damaged transplants. For example, the heterogeneity seen on virtually all MRI measurements was probably caused by scattered hemorrhages, which usually do not occur in earlier stages allograft nephropathy. The histological lesions that are routinely seen in less damaged transplants, such as fibrosis and inflammation, were present in an extreme form that is usually not seen in a functioning graft. However, the changes in *T*_1_, diffusion coefficients and perfusion which we found are in line with the literature on chronic kidney disease both in native kidneys and transplants and probably do reflect the final stage of a continuous process. *T*_1_, diffusion coefficients and perfusion measurements are promising non-invasive surrogate markers for severity of fibrosis and IF/TA, inflammation, and capillary rarefaction. However, for clinical diagnostic use of MRI, its lack of specificity remains a problem. The decrease in *T*_2_ we found is interesting in this context since it is expected to change differently in response to inflammation then to fibrosis, but more research in renal disease is needed to confirm this.

A methodological limitation of this study was that the explanted kidney was sectioned at only three levels in the coronal plane (ventral, dorsal and lateral) of the upper and lower poles. These sections, therefore, did not exactly match the coronal plane of the MRI scans. This might have led to underestimation of the accuracy by which MRI scans can detect histological abnormalities. During explantation, superficial damage was inflicted to the kidney which might have caused superficial hemorrhaging and consequently might have influenced histology. In addition, the damage to the graft was to an extent usually not seen in healthy grafts. Although our findings are in line with previous studies on MRI in patients with chronic kidney disease, it is unknown how they will translate to less severely damaged grafts. However, if in such an extreme case no changes in MRI parameters could be observed, changes are likely also not observed in less severe cases. Furthermore, the MRI images were compared to images and normative values obtained in healthy volunteers, as opposed to patients with well-functioning transplants which would have been more appropriate, but this was beyond the scope of this study. Kidney transplantation is known to induce physiological and microstructural changes in the graft, which can be measured with MRI [5], so a comparison to well-functioning grafts might yield slightly different results. In future studies, we aim to make a comparison between ill-functioning and relatively healthy transplant kidneys.

Currently, acquisition time of this multiparametric protocol was approximately 1 h, which might be challenging to fit in clinical timeslots. Future studies will most likely determine which acquisitions provide clinically valuable information and which acquisitions do not contribute and can, therefore, be discarded. This will shorten acquisition time.

In conclusion, this patient gave us the unique opportunity to directly compare MR images obtained shortly before explantation with whole organ histology. Important histological predictors of long-term graft survival after kidney transplantation, like IF/TA and capillary rarefaction, could be identified with a combination of multiple functional MRI techniques, which underscores the high potential of the latter for non-invasive acquisition of valuable information for clinical decision-making.

## Electronic supplementary material

Below is the link to the electronic supplementary material.

Supplementary file1 (DOCX 725 kb)
