# Supplementary material for: Validation of multiparametric MRI by histopathology after nephrectomy: a case study
Source: MAGMA. 2020 Sep 20;34(3):377–87. doi: 10.1007/s10334-020-00887-9 (PMC8154819; doi:10.1007/s10334-020-00887-9)
Supplement: Supplementary file 1 — Supplementary file1 (DOCX 725 kb) [file 10334_2020_887_MOESM1_ESM.docx]

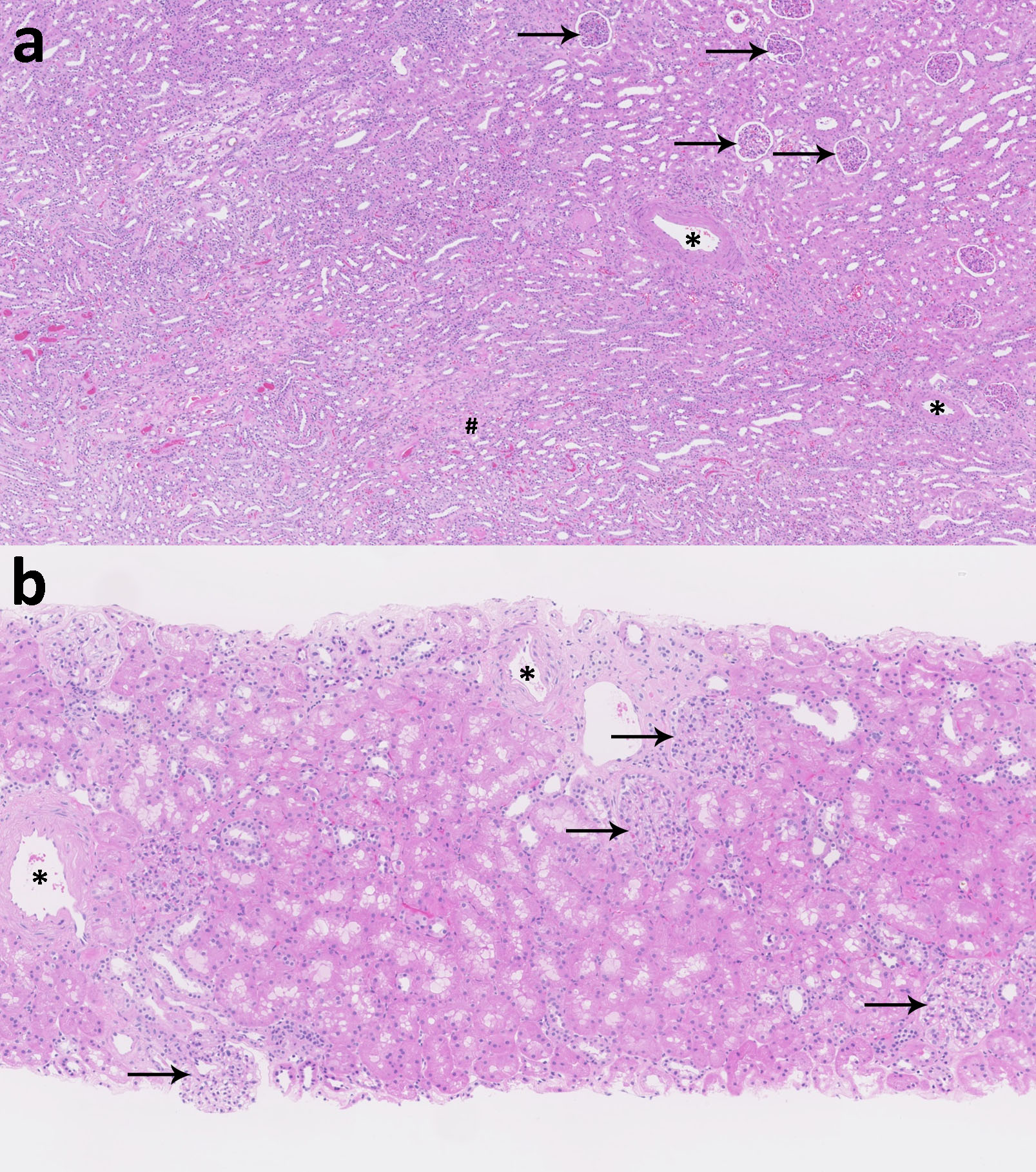


**Supplemental Figure 1** Normal histology of kidney specimens. Examples of relatively unaffected kidney tissue from (a) non-tumorous kidney of a nephrectomy specimen from a patient with renal cell carcinoma and (b) a protocol biopsy three months after transplantation. No areas of inflammation or hemorrhage are present. Shown are non-sclerosed glomeruli in the cortex (arrows), patent arteries (*) and normal medulla (#).
